# The Octopus Sign—A New HRCT Sign in Pulmonary Langerhans Cell Histiocytosis

**DOI:** 10.3390/diagnostics12040937

**Published:** 2022-04-08

**Authors:** Alexander Poellinger, Sabina Berezowska, Jeffrey Leon Myers, Adrian Huber, Manuela Funke-Chambour, Sabina Guler, Thomas Geiser, Sergio Harari, Antonella Caminati, Maurizio Zompatori, Nicola Sverzellati

**Affiliations:** 1 Department of Diagnostic, Interventional and Pediatric Radiology, University Hospital of Bern, University of Bern, 3010 Bern, Switzerland; adrian.huber@insel.ch; 2 Department of Laboratory Medicine and Pathology, Institute of Pathology, Lausanne University Hospital, University of Lausanne, Rue du Bugnon 25, 1011 Lausanne, Switzerland; sabina.berezowska@chuv.ch; 3 Department of Pathology, University of Michigan Hospital, Ann Arbor, MI 48109, USA; myerjeff@med.umich.edu; 4 Department of Pulmonary Medicine, University Hospital of Bern, University of Bern, 3010 Bern, Switzerland; manuela.funke-chambour@insel.ch (M.F.-C.); sabina.guler@insel.ch (S.G.); thomas.geiser@insel.ch (T.G.); 5 Department of Medicine, Division of Internal Medicine, Ospedale San Giuseppe MultiMedica IRCCS, University of Milan, 20133 Milan, Italy; sergio@sergioharari.it (S.H.); maurizio.zompatori@unibo.it (M.Z.); 6 U.O. di Pneumologia e Terapia Semi-Intensiva Respiratoria, Servizio di Fisiopatologia Respiratoria e Emodinamica Polmonare, Ospedale San Giuseppe, MultiMedica IRCCS, University of Milan, 20133 Milan, Italy; antonella.caminati@multimedica.it; 7 Scienze Radiologiche, Department of Medicine and Surgery, Padiglione Barbieri, University of Parma, V. Gramsci 14, 43124 Parma, Italy; nicola.sverzellati@unipr.it

**Keywords:** pulmonary Langerhans cell histiocytosis (PLCH), high-resolution computed tomography (HRCT), cystic lung disease, nodular lung disease, pathology

## Abstract

*Background*: Fibrosis in pulmonary Langerhans cell histiocytosis (PLCH) histologically comprises a central scar with septal strands and associated airspace enlargement that produce an octopus-like appearance. The purpose of this study was to identify the octopus sign on high-resolution computed tomography (HRCT) images to determine its frequency and distribution across stages of the disease. *Methods*: Fifty-seven patients with confirmed PLCH were included. Two experienced chest radiologists assessed disease stages as early, intermediate, or late, as well as the lung parenchyma for nodular, cystic, or fibrotic changes and for the presence of the octopus sign. Statistical analysis included Cohen’s kappa for interrater agreement and Fisher’s exact test for the frequency of the octopus sign. *Results*: Interobserver agreement was substantial for the octopus sign (kappa = 0.747). Significant differences in distribution of the octopus sign between stages 2 and 3 were found with more frequent octopus signs in stage 2 and fewer in stage 3. In addition, we only found the octopus sign in cases of nodular und cystic lung disease. *Conclusions*: The octopus sign in PLCH can be identified not only on histological images, but also on HRCT images. Its radiological presence seems to depend on the stage of PLCH.

## 1. Introduction

Pulmonary Langerhans cell histiocytosis (PLCH) is an interstitial lung disease with unknown etiology that primarily affects young adults in the age group of 20 to 40 years [1,2,3]. It is considered a rare disease, and the exact incidence and prevalence have not been fully investigated [4]. The strongest risk factor for the development of this disease in adults is smoking, which is reported in at least 90% of patients with adult PLCH [5]. Patients present with nonproductive cough, dyspnea, chest pain, fatigue, and weight loss [1,6,7]. Pneumothorax is the initial symptom of PLCH in 15–20% of cases; 16–36% of patients are asymptomatic at presentation [8].

The high-resolution computed tomography (HRCT) appearance of PLCH varies with a nodular, a nodular–cystic, a predominant cystic, or a fibrotic pattern [9,10]. At early stages, i.e., in patients with symptom onset within the last 6 months, a nodular pattern predominates. Later, these nodules cavitate and form thick-walled cysts [10]. The pattern changes into thin-walled cysts, often with bizarre shapes. Brauner et al. observed confluent cysts to be the dominant feature when disease was present for longer than 3 years, leading to fibrobullous destruction and a quasi-emphysematous appearance [9,10,11].

In some cases, PLCH is difficult to differentiate from other cystic diseases on HRCT, such as lymphangioleiomyomatosis, lymphoid interstitial pneumonia (LIP), and Birt–Hogg–Dubé disease, the latter two having less differential overlap with PLCH.

Histologically, PLCH presents with nodular lesions containing Langerhans cells. These cells can be found predominantly in the peribronchiolar interstitium, where they form sheets and ill-defined clusters resembling granulomas. Langerhans cells may also infiltrate the bronchiolar epithelium and spill into peribronchiolar air spaces. As lesions mature, the cellular infiltrate is replaced by collagen fibrosis that begins centrally and extends peripherally into contiguous peribronchiolar alveolar septa, leading to traction and dilatation of bronchioles. In the end, this pattern of fibrosis is accompanied by a distinctive pattern of paracicatricial airspace enlargement (“scar emphysema”), accounting for the prevalence of irregularly shaped cysts in late, fibrotic disease.

These cysts often exhibit small septal fibrotic strands emanating from a central bronchiolocentric scar that form a typical appearance resembling a star or an octopus. These octopus-like lesions are often several millimeters in diameter.

This histologic development corresponds well with the appearance on HRCT. In the early stages of the disease, the nodular pattern is predominantly followed by a pattern with cavitating nodules and thick-walled cysts to thin-walled cysts and finally fibrosis [10,11]. Since the “octopus” measures several millimeters on histological images, it is expected to be visible on HRCT as well. PLCH can be a good mimicker, and the identification of a new HRCT sign for PLCH would be of utmost importance to improve the differential diagnosis with either nodular or cystic lung disorders. In particular, the new sign could help to differentiate between certain stages of LCH and centrilobular emphysema, as these are difficult to separate.

The purpose of this study was to identify the octopus sign on HRCT images of PLCH patients in order to determine its frequency, overall and at different stages of the disease.

## 2. Materials and Methods

Fifty-seven patients from three institutions (Scienze Radiologiche, Academic Hospital of Parma, Ospedale S. Orsola-Malpighi of Bologn, IRCCS Mutimedica of Milano) with PLCH confirmed by histology or diagnosed on the basis of clinical and radiological findings were included. Two experienced chest radiologists (R1 and R2) with 19 and 17 years of experience analyzed HRCTs of the lung performed in end-inspiration.

No well-defined stages exist radiologically or histologically. In accordance with both the radiological and the histological literature, which often refers to three stages, we defined the stages in HRCT as follows: early stage, where predominant features are nodules, cavitating nodules, and thick-walled cysts [9,10,12], and no thin-walled cysts or fibrotic changes are found; intermediate stage, where a mix of thick- and thin-walled cysts, cavitating nodules, and bizarre shaped cysts is found [5,9]; late stage, where thin-walled cysts and coalescent cysts are found, leading to fibro bullous destruction of the lung [11,13,14]. Both radiologists were asked to assess the stage of the disease as early, intermediate, or late (stages 1 to 3).

Both readers also analyzed the lung parenchyma for “nodular”, “cystic”, “nodular and cystic”, and “fibrotic changes”. Nodules were defined following the Fleischner glossary as “rounded or irregular opacities, well or poorly defined” [15], while cysts were defined as “round parenchymal lucencies or low-attenuating areas with a well-defined interface with the normal lung. Cysts following the Fleischner glossary are usually thin-walled (<2 mm). In PLCH, we differentiate between thin-walled cysts and thick-walled cysts at a threshold wall thickness of 2 mm. Fibrotic changes were defined as areas with architectural distortion, i.e., abnormal displacement of bronchi, vessels, fissures, or septa and the formation of fibrotic scars surrounded by distorted and enlarged air spaces [11,13].

The octopus sign was defined as a localized area with increased lung transparency with linear opacifications that were arranged in a similar way to the tentacles of an octopus. The linear opacifications are thicker than the fine lines found in centrilobular emphysema and sometimes form nodular or spindle-shaped protrusions. The lungs were examined closely for the octopus sign with radiologists stating a degree of certainty that one or more octopus signs were present (certainty: 1 = low, 2 = medium, 3 = high). Finally, the lobes and the location of the “octopus” in the core or the peel of the lung were recorded.

Statistical analysis was carried out using Cohen’s kappa for interrater agreement. To compare frequencies of the octopus sign across the different PLCH stages and across the nodular, cystic, and fibrotic patterns, contingency tables and Fisher’s exact test were used. For this analysis, both readers in consensus redefined the stages in six cases where the stages initially differed between readers. For the Fisher’s exact test, a two-sided *p*-value of 0.05 or smaller was considered statistically significant.

## 3. Results

### 3.1. HRCT Findings

On HRCT, nodular disease was present in the majority of cases (R1: 42/57, 74% and R2: 40/57, 70%). Cystic disease was present in almost all cases (R1: 55/57, 96% and R2: 56/57, 98%). Nodular and cystic disease combined was present in most cases (R1: 40/57, 70%; R2: 39/57, 68%). There were two (R1) and one case (R2) of pure nodular, and 15 (R1) and 16 (R2) pure cystic cases out of the 57 cases. R1 classified 23 cases as fibrotic, while R2 classified 26 cases as fibrotic.

The readers (R1 and R2) classified seven and six cases as stage 1, 19 and 14 cases as stage 2, and 31 and 37 cases as stage 3, respectively.

Reader 1 detected at least one octopus sign in 32 cases: nine with a low level of certainty, eight with a medium level of certainty, and 15 with a high level of certainty. Reader 2 detected at least one octopus sign in a total of 21 cases: three with a low level of certainty, eight with a medium level of certainty, and 10 with a high level of certainty.

### 3.2. Interobserver Agreement

There was considerable inter-reader agreement (kappa = 0.747) in identifying the octopus sign with a certainty level of 3 (high) (Table 1). Interobserver agreement was fair (kappa = 0.394) when all levels of certainty were included.

There was almost perfect interobserver agreement for the presence of nodular lung disease (kappa = 0.913), substantial agreement for cystic lung disease (kappa = 0.659), almost perfect agreement for the presence of fibrotic lung disease (kappa = 0.822), and almost perfect agreement for the presence of nodular and cystic lung disease (kappa = 0.959). Agreement on the different PLCH stages was substantial (kappa = 0.809).

### 3.3. Octopus Sign for Different Stages and Different Lung Patterns

Significant differences in the distribution of the octopus sign between stages 2 and 3 were found for both readers (after consensus), with more octopus signs observed in stage 2 and fewer in stage 3 (Table 2). No octopus signs were found for pure nodular (R1: 0/2; R2: 0/1) or pure cystic disease (R1: 0/15; R2: 0/16). For the occurrence of both nodular and cystic disease, 15/40 (38%) and 10/39 (26%) octopus signs were detected by R1 and R2, respectively. Both readers detected the octopus sign in only one case with fibrotic lung disease.

## 4. Discussion

**Figure 1:**
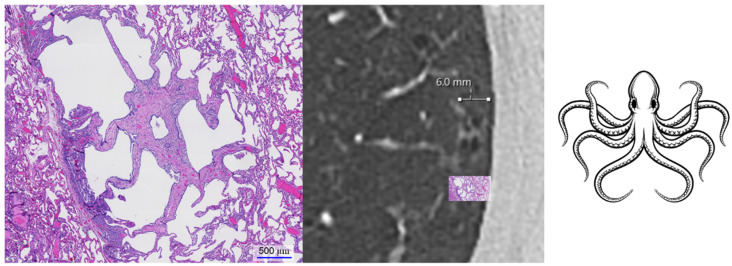
Correlation of the “octopus sign” on histology and HRCT. The histological specimen shows a fibrotic Langerhans cell histiocytosis lesion with a central, peribronchiolar fibrosis extending into the periphery, resulting in architectural distortion with traction emphysema (hematoxylin and eosin staining). The septal extensions of the lesion resemble tentacles of an octopus. The HRCT of the same patient also showed lesions that were similar in shape to an octopus.

We identified a previously undescribed HRCT finding in patients with PLCH for which we propose the term octopus sign (Figure 1). PLCH is a smoking-related lung disease with still unclear pathogenesis. Various molecular mechanisms have been identified that underlie the pathogenesis and progression of the disease [5]. One of the characteristics of early, cellular PLCH is an accumulation of a large number of CD1a^+^ Langerhans cells in loosely formed bronchiolocentric sheets and ill-defined clusters [5,16]. With time, the cellular infiltrates are replaced by scaring fibrosis, leading to traction and, thus, architectural distortion of the lung parenchyma [16,17,18]. These changes are well detectable in histopathology, as they form stellate or octopus-like lesions [13,18]. Due to the comparatively large size of several millimeters in diameter, we hypothesized that these lesions should be visible on HRCTs.

**Figure 2:**
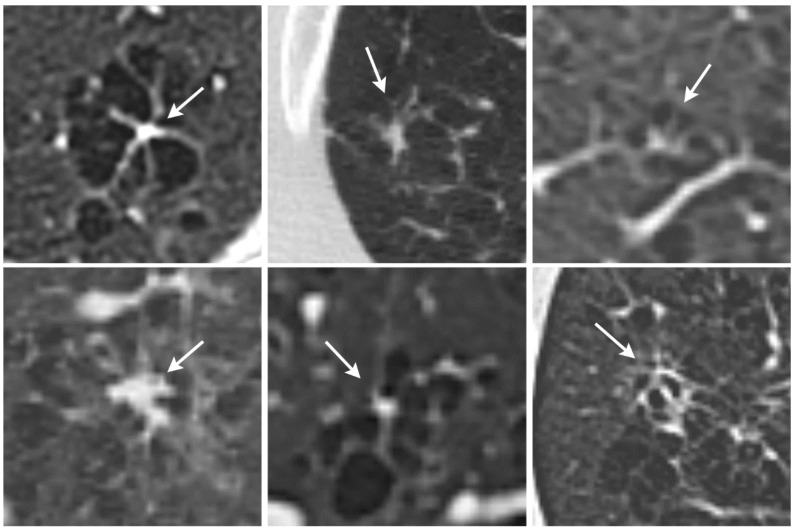
The octopus sign (arrows) in six different patients with Langerhans cell histiocytosis (stage 2).

For our study, we classified PLCH into three stages on the basis of HRCT appearance: stage 1 for early, stage 2 for intermediate, and stage 3 for late manifestations of the disease. Most octopus signs were found in stage 2 PLCH patients (Figure 2). There was a significantly different distribution of the octopus sign across patients with nodular, cystic, nodular–cystic, and fibrotic disease. No octopus sign was found in pure nodular or pure cystic lung disease, while the vast majority of octopus signs were detected in cases with simultaneous nodular and cystic lung disease. The octopus sign was found in only one case of fibrotic lung disease. These findings indicate that the radiological octopus sign is most likely to be found in intermediate stages in the natural history of PLCH.

Since the histological octopus sign represents a fibrotic lesion, we would intuitively expect more radiological octopus signs in fibrotic cases. We suspect that the octopus sign is primarily observed in incipient fibrosis, whereas it is not readily apparent in more advanced cases with significant architectural distortions. At this stage, the octopus might be replaced by paracicatricial airspace enlargement with “bizarrely shaped cysts”. Considering the octopus sign a sign of early fibrosis, the question arises whether PLCH is reversible at a stage when an octopus sign appears on HRCT. Further longitudinal studies are needed to answer this question.

**Figure 3:**
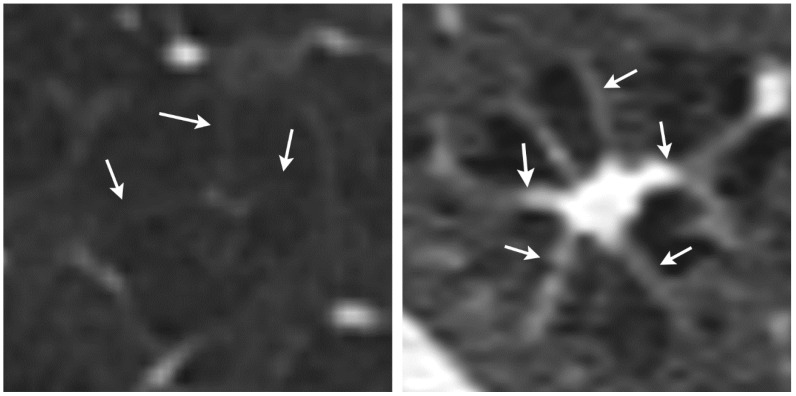
Comparison between centrilobular emphysema (**left**) and Langerhans cell histiocytosis (**right**). The strands (arrows) pointing to the center of the lesion in centrilobular emphysema are thinner than those in PLCH.

Although the combination of nodular and cystic lung disease and the bizarre form of the cysts for PLCH on HRCT are almost pathognomonic in some cases, the findings in other cases of PLCH are sometimes difficult to distinguish from other cystic lung diseases. One differential diagnosis to PLCH is centrilobular emphysema [14]. PLCH can form cystic lesions that resemble those seen in centrilobular emphysema. In addition, centrilobular emphysema may have a similar appearance to an octopus sign in that it often presents with some fine strands toward the centrilobular artery and bronchiole. We found that the octopus sign in PLCH has thicker strands toward the center, which forms the body of the octopus (Figure 3). Currently, we have only compared the octopus sign in PLCH with a few selected cases of centrilobular emphysema, and a diagnostic test study needs to be conducted to evaluate the discriminative performance of the octopus sign. Since both PLCH and centrilobular emphysema are smoking-related lung diseases, the octopus sign might contribute to the differentiation between centrilobular emphysema and PLCH. This differential diagnosis is of prognostic importance as PLCH can lead to fibrotic lung disease with unfavorable outcome [19].

Another diagnostic challenge, mainly in young female smokers, is the differentiation between PLCH and lymphangioleiomyomatosis (LAM) [20]. LAM usually presents with only cysts; however, in rare cases, cysts and nodules may be present [21]. Having another sign to rely on in the radiological diagnosis of PLCH might help for differentiation between the two diseases.

Typically, cystic lesions in Birt–Hogg–Dubé disease and LIP are quite distinct from cystic lesions in PLCH. However, especially when there are concomitant smoking-related lesions, it may be difficult to distinguish between PLCH and either disease. Again, the octopus sign may be helpful for differential diagnosis.

The natural history of PLCH is variable and unpredictable in the individual patient. After smoking cessation, some patients with PLCH have reversible or stable disease [2,8], whereas other patients develop progressive lung disease, often associated with airflow limitation and pulmonary vascular disease [4,22,23]. The prognostic importance of the octopus sign needs to be examined in longitudinal follow-up studies.

On HRCT, we obtain submillimeter resolution at best; in histology, spatial resolution is usually less than 10 um. Bridging the gap between macroscopic and microscopic patterns of disease could facilitate a morphological understanding of disease.

## 5. Conclusions

The octopus sign in PLCH can be identified not only on histological images, but also on HRCT images. Its radiological presence seems to depend on the stage of PLCH. Further studies are needed to determine the diagnostic and prognostic value of this new sign in PLCH.

## Figures and Tables

**Table 1:** Interrater agreement for different conditions.

Condition	Kappa
Octopus Sign (level of certainty 3)	0.747
Octopus Sign (level of certainty 1 to 3)	0.394
Stages (all stages)	0.809
Nodular lung disease	0.913
Cystic lung disease	0.659
Cystic and nodular lung disease	0.959
Fibrotic lung disease	0.822

**Table 2:** Frequency of the octopus sign across the different PLCH stages and across the nodular, cystic, and fibrotic patterns for both readers and for a high certainty of the octopus sign.

		Reader 1: Octopus Present	Fisher’s Exact Test *p*-Value	Reader 2: Octopus Present	Fisher’s Exact Test *p*-Value
**Stage 1 vs. 2**	1	2/7 (29%)	0.381	1/7 (14%)	0.350
	2	8/15 (53%)		6/15 (40%)	
**Stage 2 vs. 3**	2	8/15 (53%)	0.011 *	6/15 (40%)	0.015 *
	3	5/35 (14%)		3/35 (9%)	
**Stage 1 vs. 3**	1	2/7 (29%)	0.579	1/7 (14%)	0.532
	3	5/35 (14%)		3/35 (9%)	
**Pure** **Nodular**	yes	0/2 (0%)	1.000	0/1 (0%)	1.000
	no	15/55 (27%)		10/56 (18%)	
**Pure Cystic**	yes	0/15 (0%)	0.006 *	0/16 (0%)	0.048 *
	no	15/42 (36%)		10/41 (24%)	
**Nodular + Cystic**	yes	15/40 (38%)	0.002 *	10/39 (26%)	0.022 *
	no	0/17 (0%)		0/18 (0%)	
**Fibrotic**	yes	1/23 (4%)	0.002 *	1/26 (4%)	0.016 *
	no	14/34 (41%)		9/31 (29%)	

* Statistically significant.

## Data Availability

Supporting data are available for review from the corresponding author.
